# Crystal structure of (*Z*)-2-hy­droxy-4-methyl-*N*′-(4-oxo-1,3-thia­zolidin-2-yl­idene)benzohydrazide trihydrate

**DOI:** 10.1107/S1600536814023356

**Published:** 2014-10-29

**Authors:** Peijuan Li, Zizhen Kang, Xin Fan, Longfei Jin

**Affiliations:** a College of Chemistry and Material Science, South-Central University for Nationalities, Wuhan 430074, People’s Republic of China

**Keywords:** crystal structure, benzohydrazide, 1,3-thia­zol­idene, hydrogen bonding, biological activity

## Abstract

In the title compound, C_11_H_11_N_3_O_3_S·3H_2_O, the non-H atoms of the main mol­ecule are approximately planar, with an r.m.s. deviation of 0.030 Å. There is a bifurcated intra­molecular N—H⋯(O,S) hydrogen bond present forming *S*(6) and *S*(5) ring motifs. In the crystal, O—H⋯O and N—H⋯O hydrogen bonds link the molecules into a three-dimensional network.

## Related literature   

For the biological activities of thia­zolidin-4-one compounds, see: Jain *et al.* (2012 ▶); Verma & Saraf (2008 ▶); Singh *et al.* (1981 ▶). For the synthesis, see: Brown (1961 ▶).
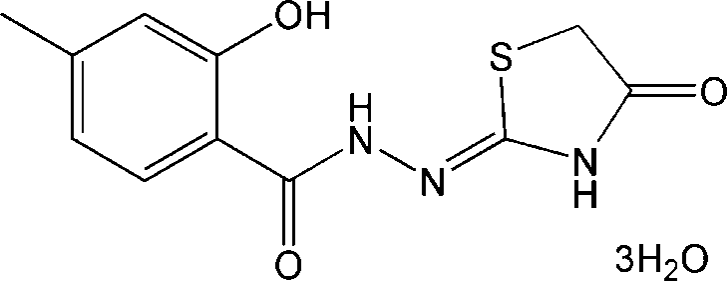


## Experimental   

### Crystal data  

C_11_H_11_N_3_O_3_S·3H_2_O
*M* *_r_* = 319.33
Triclinic, 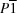

*a* = 7.3739 (12) Å
*b* = 8.5110 (13) Å
*c* = 12.493 (2) Å
α = 103.047 (2)°
β = 101.385 (2)°
γ = 92.532 (2)°
*V* = 745.5 (2) Å^3^
*Z* = 2
Mo *K*α radiation
μ = 0.25 mm^−1^
*T* = 296 K
0.21 × 0.20 × 0.18 mm

### Data collection  

Bruker APEXII CCD diffractometer
Absorption correction: multi-scan (*SADABS*; Sheldrick, 1996 ▶) *T*_min_ = 0.950, *T*_max_ = 0.957
5460 measured reflections
2731 independent reflections
2266 reflections with *I* > 2σ(*I*)
*R*_int_ = 0.020

### Refinement  

*R*[*F* ^2^ > 2σ(*F* ^2^)] = 0.052
*wR*(*F* ^2^) = 0.154
*S* = 1.10
2731 reflections
191 parameters
H-atom parameters constrained
Δρ_max_ = 0.53 e Å^−3^
Δρ_min_ = −0.44 e Å^−3^

### 

Data collection: *APEX2* (Bruker, 2001 ▶); cell refinement: *SAINT* (Bruker, 2001 ▶); data reduction: *SAINT*; program(s) used to solve structure: *SHELXS97* (Sheldrick, 2008 ▶); program(s) used to refine structure: *SHELXL2014* (Sheldrick, 2008 ▶); molecular graphics: *SHELXTL* (Sheldrick, 2008 ▶) and *PLATON* (Spek, 2009 ▶); software used to prepare material for publication: *SHELXTL*.

## Supplementary Material

Crystal structure: contains datablock(s) global, I. DOI: 10.1107/S1600536814023356/lh5733sup1.cif

Structure factors: contains datablock(s) I. DOI: 10.1107/S1600536814023356/lh5733Isup2.hkl

Supporting information file. DOI: 10.1107/S1600536814023356/lh5733Isup3.cml

**Fig. 1.**
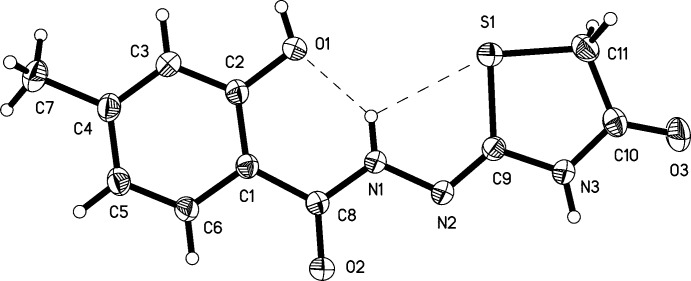
The molecular structure of (I), showing 30% probability displacement ellipsoids. The solvent water molecules have been omitted for clarity. Dashed lines indicate hydrogen bonds.

. DOI: 10.1107/S1600536814023356/lh5733fig1.tif
The mol­ecular structure of (I), showing 30% probability displacement ellipsoids. The solvent water mol­ecules have been omitted for clarity. Dashed lines indicate hydrogen bonds.

**Fig. 2.**
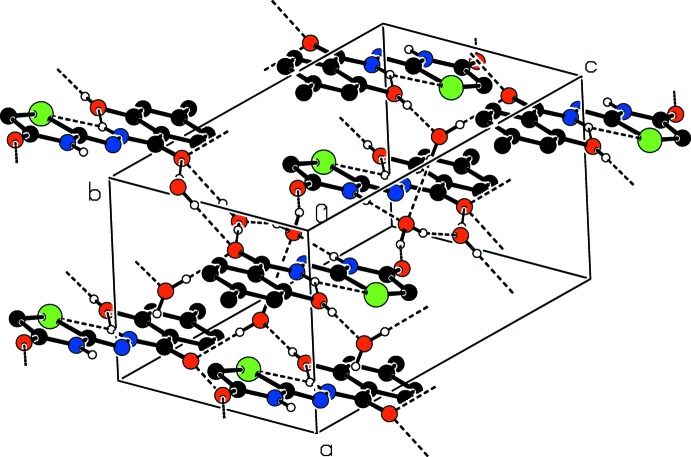
Part of the crystal structure of (I) showing hydrogen bonds as dashed lines.

. DOI: 10.1107/S1600536814023356/lh5733fig2.tif
Part of the crystal structure of (I) showing hydrogen bonds as dashed lines.

CCDC reference: 1030606

Additional supporting information:  crystallographic information; 3D view; checkCIF report

## Figures and Tables

**Table 1:** Hydrogen-bond geometry (, )

*D*H*A*	*D*H	H*A*	*D* *A*	*D*H*A*
O1H1*O*O5^i^	0.84	1.83	2.655(3)	167
N1H1*A*S1	0.86	2.51	2.941(2)	112
N1H1*A*O1	0.86	1.92	2.609(3)	137
N3H3*B*O4	0.86	1.89	2.739(4)	170
O4H4*OA*O3^ii^	0.84	2.10	2.813(4)	143
O4H4*OB*O6	0.81	1.81	2.587(4)	161
O5H5*OA*O2^iii^	0.84	2.02	2.864(3)	178
O5H5*OB*O4	0.84	2.40	3.239(6)	180
O6H6*OA*O2^iv^	0.84	2.11	2.947(4)	180
O6H6*OB*O2	0.84	2.02	2.862(3)	180

Symmetry codes: (i) 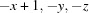; (ii) 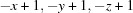; (iii) 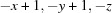; (iv) 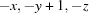.

## supplementary crystallographic information

### S1. Comment

4-Thiazolidinones are compounds which have a sulfur atom at position 1, a nitrogen atom at position 3 and a carbonyl group at position 4. Derivatives of 4-thiazolidinone exhibit prominent biological activites such as antibacterial, antifungal, antitubercular, anticancer, antiinflammtory, analgesic, anticonvulsant, antidepressant, antiviral/anti-HIV, antidiabetic, muscarinic receptor 1 agonist, FSH receptor agonist, trypanocidal (anti-epimastigote) and antiarrhythmic activity (Jain, *et al.*, 2012; Verma & Saraf, 2008; Singh *et al.*, 1981). Several hydrogen bond acceptor sites exist in these compounds, which could potentially lead to the formation of supermolecular structures. As part of our ongoing studies, the preparation and X-ray structure determination of the title compound, (I), was undertaken.

In the title molecule (Fig. 1) the bond lengths show normal ranges of values. The non-hydrogen atoms of the main molecule are approximately planar with an r.m.s. deviation of 0.030Å. There is a bifurcated intramolecular N—H···(O,S) hydrogen bond present forming S(6) and S(5) ring motifs. In the crystal, O—H···O and N—H···O hydrogen bonds form a three-dimensional network (Fig. 2).

### S2. Experimental

The synthesis followed the prodecures of Brown (1961). 4-(4-Methyl salicyloyl) thiosemicarbazide (2.25 g, 0.01 mol), ethyl bromoacetate (3.34 g, 0.02 mol) and 50 ml of ethyl alcohol were added to a round-bottom flask. The mixture was stirred for 10 minutes, then slowly warmed to boiling and stirred for 8 h. After cooling to room temperature, 40 ml of water were added and the reaction mixture was left for 12 h. The resulting precipitate was filtered and recrystallized with ethyl alcohol to give 2.30 g of the title compound. Single crystals suitable for X-ray diffraction analysis were growned by slow evaporation of a solution of the title compound in methanol/water/ether (20:7:5) at room temperature.

### S3. Refinement

H atoms bonded to C and N atoms were placed in calculated positions and included in a riding-model approximation with C—H = 0.93–0.97Å, N—H = 0.86Å and U_iso_(H)=1.2U_eq_(C,N) or 1.5U_eq_(C_methyl_). The hydroxyl H atom was placed in an 'as found' position and refined as riding with U_iso_(H)=1.5U_eq_(O). The H atoms bonded to the solvent water molecules were included in positions which gave the most sensible and consistent hydrogen bond interactions and were refined as riding with U_iso_(H)=1.5U_eq_(O).

### Crystal data

**Table:**

C_11_H_11_N_3_O_3_S·3H_2_O	*Z* = 2
*M**_r_* = 319.33	*F*(000) = 336
Triclinic, *P*1	*D*_x_ = 1.423 Mg m^−^^3^
*a* = 7.3739 (12) Å	Mo *K*α radiation, λ = 0.71073 Å
*b* = 8.5110 (13) Å	Cell parameters from 2596 reflections
*c* = 12.493 (2) Å	θ = 2.5–31.1°
α = 103.047 (2)°	µ = 0.25 mm^−^^1^
β = 101.385 (2)°	*T* = 296 K
γ = 92.532 (2)°	Block, yellow
*V* = 745.5 (2) Å^3^	0.21 × 0.20 × 0.18 mm

### Data collection

**Table:**

Bruker APEXII CCD diffractometer	2266 reflections with *I* > 2σ(*I*)
Radiation source: fine-focus sealed tube	*R*_int_ = 0.020
φ and ω scans	θ_max_ = 25.5°, θ_min_ = 1.7°
Absorption correction: multi-scan (*SADABS*; Sheldrick, 1996)	*h* = −8→8
*T*_min_ = 0.950, *T*_max_ = 0.957	*k* = −10→10
5460 measured reflections	*l* = −14→15
2731 independent reflections	

### Refinement

**Table:**

Refinement on *F*^2^	0 restraints
Least-squares matrix: full	Hydrogen site location: mixed
*R*[*F*^2^ > 2σ(*F*^2^)] = 0.052	H-atom parameters constrained
*wR*(*F*^2^) = 0.154	*w* = 1/[σ^2^(*F*_o_^2^) + (0.0721*P*)^2^ + 0.5146*P*] where *P* = (*F*_o_^2^ + 2*F*_c_^2^)/3
*S* = 1.10	(Δ/σ)_max_ < 0.001
2731 reflections	Δρ_max_ = 0.53 e Å^−^^3^
191 parameters	Δρ_min_ = −0.44 e Å^−^^3^

### Special details

**Table:**

Geometry. All esds (except the esd in the dihedral angle between two l.s. planes) are estimated using the full covariance matrix. The cell esds are taken into account individually in the estimation of esds in distances, angles and torsion angles; correlations between esds in cell parameters are only used when they are defined by crystal symmetry. An approximate (isotropic) treatment of cell esds is used for estimating esds involving l.s. planes.

### Fractional atomic coordinates and isotropic or equivalent isotropic displacement parameters (Å^2^)

**Table:**

	*x*	*y*	*z*	*U*_iso_*/*U*_eq_	
S1	0.43631 (10)	−0.07307 (8)	0.18232 (6)	0.0437 (2)	
O1	0.2052 (3)	−0.2057 (2)	−0.11504 (16)	0.0516 (5)	
H1O	0.2170	−0.3043	−0.1393	0.077*	
O2	0.1220 (3)	0.2836 (2)	−0.06337 (16)	0.0554 (6)	
O3	0.6060 (3)	0.2112 (3)	0.47760 (17)	0.0650 (6)	
N1	0.2365 (3)	0.0858 (3)	0.01408 (17)	0.0383 (5)	
H1A	0.2525	−0.0154	0.0060	0.046*	
N2	0.3037 (3)	0.1936 (2)	0.11730 (17)	0.0394 (5)	
N3	0.4615 (3)	0.2237 (3)	0.30031 (18)	0.0424 (5)	
H3B	0.4541	0.3266	0.3168	0.051*	
C1	0.0805 (3)	0.0165 (3)	−0.1811 (2)	0.0348 (5)	
C2	0.1095 (4)	−0.1485 (3)	−0.2005 (2)	0.0385 (6)	
C3	0.0424 (4)	−0.2507 (3)	−0.3061 (2)	0.0459 (7)	
H3A	0.0628	−0.3597	−0.3176	0.055*	
C4	−0.0540 (4)	−0.1944 (3)	−0.3946 (2)	0.0444 (6)	
C5	−0.0822 (4)	−0.0295 (3)	−0.3762 (2)	0.0436 (6)	
H5A	−0.1456	0.0114	−0.4345	0.052*	
C6	−0.0159 (4)	0.0715 (3)	−0.2714 (2)	0.0394 (6)	
H6A	−0.0360	0.1806	−0.2603	0.047*	
C7	−0.1260 (5)	−0.3079 (4)	−0.5088 (3)	0.0658 (9)	
H7A	−0.1713	−0.4104	−0.4996	0.099*	
H7B	−0.0272	−0.3230	−0.5489	0.099*	
H7C	−0.2251	−0.2622	−0.5504	0.099*	
C8	0.1471 (4)	0.1390 (3)	−0.0727 (2)	0.0365 (6)	
C9	0.3903 (3)	0.1303 (3)	0.1930 (2)	0.0361 (6)	
C10	0.5429 (4)	0.1475 (4)	0.3782 (2)	0.0450 (6)	
C11	0.5482 (4)	−0.0303 (4)	0.3291 (2)	0.0484 (7)	
H11A	0.4837	−0.0940	0.3678	0.058*	
H11B	0.6758	−0.0574	0.3373	0.058*	
O4	0.4027 (6)	0.5448 (4)	0.3300 (3)	0.1451 (18)	
H4OA	0.3560	0.5885	0.3846	0.218*	
H4OB	0.3221	0.5540	0.2778	0.218*	
O5	0.7047 (5)	0.5074 (3)	0.1718 (3)	0.1071 (12)	
H5OA	0.7543	0.5677	0.1388	0.161*	
H5OB	0.6264	0.5168	0.2128	0.161*	
O6	0.1984 (7)	0.5417 (4)	0.1356 (3)	0.160 (2)	
H6OA	0.1074	0.5918	0.1148	0.241*	
H6OB	0.1766	0.4661	0.0771	0.241*	

### Atomic displacement parameters (Å^2^)

**Table:**

	*U*^11^	*U*^22^	*U*^33^	*U*^12^	*U*^13^	*U*^23^
S1	0.0523 (4)	0.0375 (4)	0.0412 (4)	0.0101 (3)	0.0083 (3)	0.0095 (3)
O1	0.0767 (14)	0.0345 (10)	0.0386 (10)	0.0106 (9)	−0.0014 (9)	0.0093 (8)
O2	0.0862 (15)	0.0350 (10)	0.0397 (11)	0.0137 (10)	0.0006 (10)	0.0076 (8)
O3	0.0801 (15)	0.0747 (15)	0.0328 (11)	0.0121 (12)	−0.0027 (10)	0.0094 (10)
N1	0.0508 (13)	0.0315 (10)	0.0296 (11)	0.0045 (9)	0.0029 (9)	0.0056 (8)
N2	0.0496 (13)	0.0344 (11)	0.0309 (11)	0.0037 (9)	0.0039 (9)	0.0050 (9)
N3	0.0535 (13)	0.0363 (11)	0.0323 (11)	0.0036 (10)	0.0002 (10)	0.0058 (9)
C1	0.0363 (13)	0.0376 (13)	0.0311 (12)	0.0036 (10)	0.0081 (10)	0.0083 (10)
C2	0.0431 (14)	0.0394 (14)	0.0330 (13)	0.0032 (11)	0.0072 (11)	0.0098 (10)
C3	0.0568 (17)	0.0372 (14)	0.0395 (15)	0.0060 (12)	0.0054 (12)	0.0046 (11)
C4	0.0447 (15)	0.0492 (15)	0.0341 (14)	0.0044 (12)	0.0026 (11)	0.0044 (12)
C5	0.0424 (14)	0.0534 (16)	0.0342 (13)	0.0114 (12)	0.0046 (11)	0.0105 (12)
C6	0.0419 (14)	0.0410 (14)	0.0356 (13)	0.0098 (11)	0.0074 (11)	0.0094 (11)
C7	0.080 (2)	0.059 (2)	0.0419 (17)	0.0092 (17)	−0.0070 (15)	−0.0042 (14)
C8	0.0416 (13)	0.0350 (13)	0.0330 (13)	0.0040 (10)	0.0073 (10)	0.0089 (10)
C9	0.0383 (13)	0.0352 (13)	0.0343 (13)	0.0014 (10)	0.0081 (10)	0.0079 (10)
C10	0.0467 (15)	0.0555 (16)	0.0342 (14)	0.0067 (12)	0.0068 (12)	0.0149 (12)
C11	0.0536 (17)	0.0538 (17)	0.0426 (15)	0.0139 (13)	0.0107 (13)	0.0194 (13)
O4	0.216 (4)	0.0616 (18)	0.097 (2)	0.052 (2)	−0.072 (2)	−0.0211 (16)
O5	0.162 (3)	0.0516 (15)	0.139 (3)	0.0217 (17)	0.088 (3)	0.0362 (17)
O6	0.285 (5)	0.0653 (19)	0.073 (2)	0.074 (3)	−0.067 (3)	−0.0197 (15)

### Geometric parameters (Å, º)

**Table:**

S1—C9	1.759 (3)	C3—H3A	0.9300
S1—C11	1.803 (3)	C4—C5	1.401 (4)
O1—C2	1.357 (3)	C4—C7	1.511 (4)
O1—H1O	0.8400	C5—C6	1.376 (4)
O2—C8	1.236 (3)	C5—H5A	0.9300
O3—C10	1.224 (3)	C6—H6A	0.9300
N1—C8	1.334 (3)	C7—H7A	0.9600
N1—N2	1.387 (3)	C7—H7B	0.9600
N1—H1A	0.8600	C7—H7C	0.9600
N2—C9	1.271 (3)	C10—C11	1.504 (4)
N3—C10	1.349 (3)	C11—H11A	0.9700
N3—C9	1.382 (3)	C11—H11B	0.9700
N3—H3B	0.8600	O4—H4OA	0.8430
C1—C6	1.396 (4)	O4—H4OB	0.8119
C1—C2	1.404 (4)	O5—H5OA	0.8400
C1—C8	1.489 (3)	O5—H5OB	0.8399
C2—C3	1.388 (4)	O6—H6OA	0.8400
C3—C4	1.385 (4)	O6—H6OB	0.8400
			
C9—S1—C11	91.62 (12)	C1—C6—H6A	118.8
C2—O1—H1O	108.8	C4—C7—H7A	109.5
C8—N1—N2	120.0 (2)	C4—C7—H7B	109.5
C8—N1—H1A	120.0	H7A—C7—H7B	109.5
N2—N1—H1A	120.0	C4—C7—H7C	109.5
C9—N2—N1	114.6 (2)	H7A—C7—H7C	109.5
C10—N3—C9	117.5 (2)	H7B—C7—H7C	109.5
C10—N3—H3B	121.2	O2—C8—N1	121.3 (2)
C9—N3—H3B	121.2	O2—C8—C1	121.7 (2)
C6—C1—C2	117.5 (2)	N1—C8—C1	117.0 (2)
C6—C1—C8	117.0 (2)	N2—C9—N3	120.5 (2)
C2—C1—C8	125.5 (2)	N2—C9—S1	128.4 (2)
O1—C2—C3	120.8 (2)	N3—C9—S1	111.13 (18)
O1—C2—C1	119.0 (2)	O3—C10—N3	125.6 (3)
C3—C2—C1	120.1 (2)	O3—C10—C11	122.5 (3)
C4—C3—C2	121.7 (3)	N3—C10—C11	111.9 (2)
C4—C3—H3A	119.2	C10—C11—S1	107.76 (18)
C2—C3—H3A	119.2	C10—C11—H11A	110.2
C3—C4—C5	118.5 (2)	S1—C11—H11A	110.2
C3—C4—C7	120.8 (3)	C10—C11—H11B	110.2
C5—C4—C7	120.7 (3)	S1—C11—H11B	110.2
C6—C5—C4	119.7 (2)	H11A—C11—H11B	108.5
C6—C5—H5A	120.1	H4OA—O4—H4OB	100.2
C4—C5—H5A	120.1	H5OA—O5—H5OB	135.7
C5—C6—C1	122.5 (2)	H6OA—O6—H6OB	95.3
C5—C6—H6A	118.8		
			
C8—N1—N2—C9	−178.4 (2)	C6—C1—C8—O2	−1.6 (4)
C6—C1—C2—O1	179.0 (2)	C2—C1—C8—O2	177.2 (3)
C8—C1—C2—O1	0.2 (4)	C6—C1—C8—N1	178.9 (2)
C6—C1—C2—C3	−0.4 (4)	C2—C1—C8—N1	−2.3 (4)
C8—C1—C2—C3	−179.2 (2)	N1—N2—C9—N3	−178.8 (2)
O1—C2—C3—C4	−179.4 (3)	N1—N2—C9—S1	0.7 (4)
C1—C2—C3—C4	0.0 (4)	C10—N3—C9—N2	176.4 (2)
C2—C3—C4—C5	0.5 (4)	C10—N3—C9—S1	−3.1 (3)
C2—C3—C4—C7	−180.0 (3)	C11—S1—C9—N2	−177.6 (3)
C3—C4—C5—C6	−0.6 (4)	C11—S1—C9—N3	1.9 (2)
C7—C4—C5—C6	179.9 (3)	C9—N3—C10—O3	−177.0 (3)
C4—C5—C6—C1	0.2 (4)	C9—N3—C10—C11	2.8 (4)
C2—C1—C6—C5	0.3 (4)	O3—C10—C11—S1	178.6 (2)
C8—C1—C6—C5	179.2 (2)	N3—C10—C11—S1	−1.2 (3)
N2—N1—C8—O2	−0.1 (4)	C9—S1—C11—C10	−0.4 (2)
N2—N1—C8—C1	179.4 (2)		

### Hydrogen-bond geometry (Å, º)

**Table:**

*D*—H···*A*	*D*—H	H···*A*	*D*···*A*	*D*—H···*A*
O1—H1*O*···O5^i^	0.84	1.83	2.655 (3)	167
N1—H1*A*···S1	0.86	2.51	2.941 (2)	112
N1—H1*A*···O1	0.86	1.92	2.609 (3)	137
N3—H3*B*···O4	0.86	1.89	2.739 (4)	170
O4—H4*OA*···O3^ii^	0.84	2.10	2.813 (4)	143
O4—H4*OB*···O6	0.81	1.81	2.587 (4)	161
O5—H5*OA*···O2^iii^	0.84	2.02	2.864 (3)	178
O5—H5*OB*···O4	0.84	2.40	3.239 (6)	180
O6—H6*OA*···O2^iv^	0.84	2.11	2.947 (4)	180
O6—H6*OB*···O2	0.84	2.02	2.862 (3)	180

Symmetry codes: (i) −*x*+1, −*y*, −*z*; (ii) −*x*+1, −*y*+1, −*z*+1; (iii) −*x*+1, −*y*+1, −*z*; (iv) −*x*, −*y*+1, −*z*.
