# Supplementary figures and images for: Crystal structure of (Z)-2-hy­droxy-4-methyl-N′-(4-oxo-1,3-thia­zolidin-2-yl­idene)benzohydrazide trihydrate
Source: Acta Crystallogr Sect E Struct Rep Online. 2014 Oct 29;70(Pt 11):o1199. doi: 10.1107/S1600536814023356 (PMC4257310; doi:10.1107/S1600536814023356)

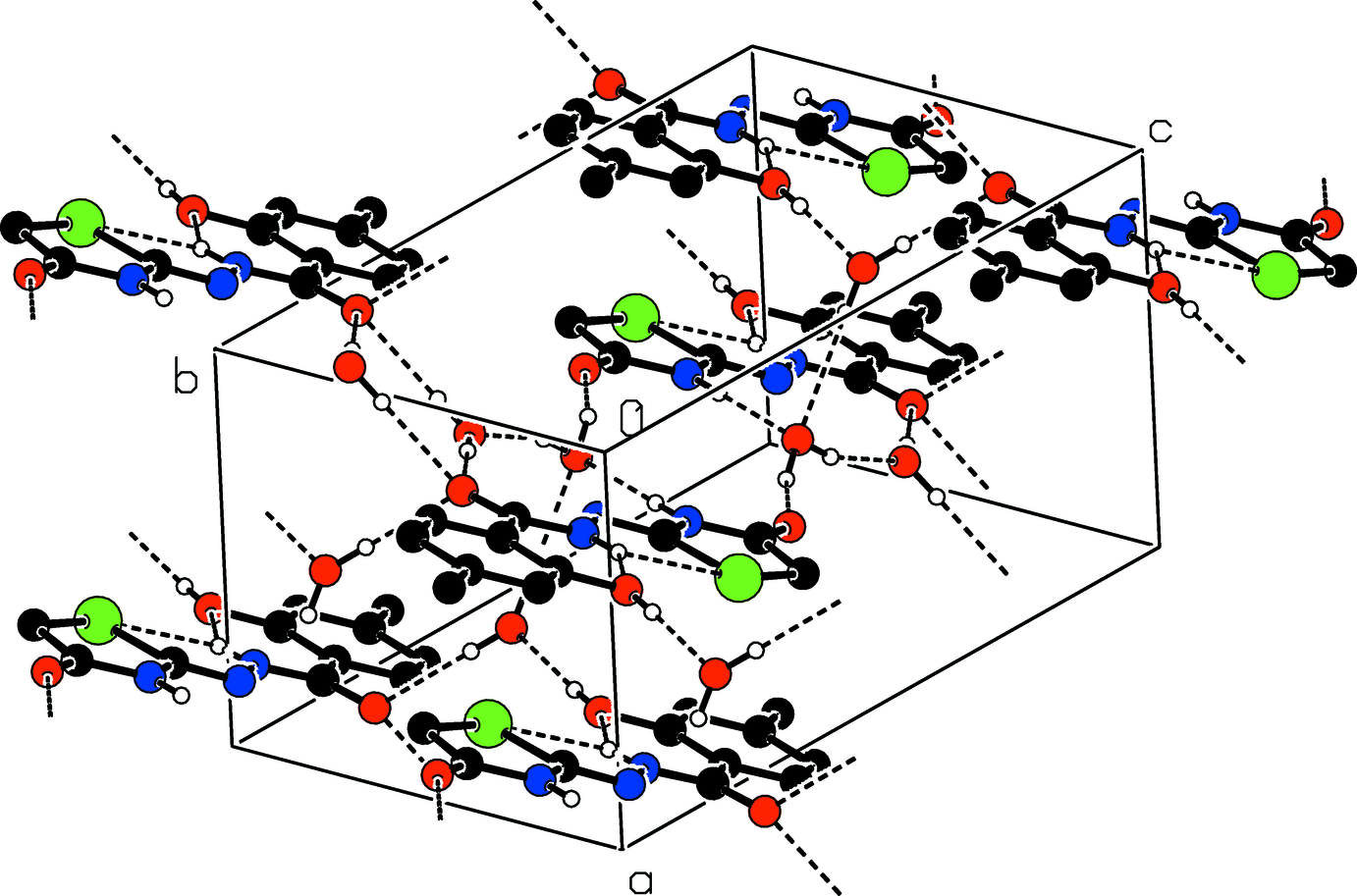

Supplement: Supplementary file 5 [file e-70-o1199-fig2.tif]
